# Association of Synthetic House-Tree-Person Drawing Test and Depression in Cancer Patients

**DOI:** 10.1155/2019/1478634

**Published:** 2019-07-28

**Authors:** Guifang Yang, Liping Zhao, Lijuan Sheng

**Affiliations:** ^1^Department of Emergency Medicine, The Second Xiangya Hospital, Central South University, China; ^2^Department of Nursing, The Second Xiangya Hospital, Central South University, China; ^3^Department of Neurology, The Second Xiangya Hospital, Central South University, China

## Abstract

**Background:**

Evidence regarding the relationship between synthetic house-tree-person (S-HTP) drawing test and depression in cancer patients is limited. The aim of this study was to explore the applicability and validity of S-HTP drawing test in cancer patients suffering from depression.

**Methods:**

As a population based cross-sectional study, 167 patients with cancer were enrolled in a hospital in China from December 2015 to December 2017. Self-edited general information questionnaire, self-rating depression scale (SDS), and the S-HTP drawing test were completed by all participants.

**Results:**

The average age of 167 selected participants was 52.92 ± 10.43 years old, and about 58.7% (98/167) of them were male. On SDS, depression rate was found in 34.1% (27/167) cancer patients. The logistic regression equation was established by using the depression drawing characteristics as the independent variables and the evaluation results from SDS as the dependent variable and 9 drawing characteristics employed in the regression equation (*χ*2 = 68.657, P < 0.001. Nagelkerke R^2^= 0.466). Correlation analysis revealed a positive correlation between S-HTP drawing test and SDS for depression state of cancer patients (p < 0.01).

**Conclusions:**

There are interrater reliability and test-retest reliability between S-HTP drawing test and SDS. The S-HTP drawing test could help in screening depression in cancer patients.

## 1. Introduction

The increase in morbidity and mortality of cancer has made it the leading cause of death in China since 2010 [1]. Currently, half of the cancer patients can expect to survive for at least 10 years because of advances in cancer treatments [2]. Meanwhile, more researchers begin to pay attention to the mental health problems such as anxiety and depression in cancer survivors [3, 4]. A meta-analysis showed that 15% of cancer patients with nonpalliative treatment were diagnosed with major depression [5]. Depression, a state of low mood and aversion to activity, can affect a person's thoughts, behavior, tendencies, feelings, and sense of well-being [6]. In cancer settings, evidence shows that depression causes serious suffering and distress, reduces participation with medical care, and potentially prolongs the duration of stay in hospital. Depression is also a significant determinant of quality of life and survival. Yet depression is often overlooked by busy cancer professionals in palliative-care and nonpalliative-care settings [5]. In previous studies, we found that the investigation of the psychological problems of cancer patients is mostly in the form of a questionnaire, such as distress thermometer [5]. However, this method is easy to cause the resistance and noncooperation of cancer patients, which may also make them alert or defensive. When the patients are unable to express their inner emotions and thoughts in words, and the subjects are unwilling to cooperate, there will be a deviation in the use of the objective scale to test the emotional state of the subjects [7, 8]. At this time, a projective test can be used as an important supplement. In psychology, a projective test is a personality test designed to let a person respond to ambiguous stimuli, presumably revealing hidden emotions and internal conflicts projected by the person into the test [7, 8]. Projective tests can enable subjects to avoid the instinctive defensive psychology, so as to obtain their inner true thoughts and emotions [9]. The synthetic house-tree-person (S-HTP) drawing test is a very important projective test.

In 1948, Buck [10] has first developed the house-tree-person drawing test that required participants to draw three subjects on three pages. In 1979, Mikami [11] developed the S-HTP drawing test, of which characteristic is the house, tree, and person which are drawn on the same sheet of paper and assessed together in relation to each other. Currently, there are many types of research using S-HTP drawing test to assess psychological functioning such as children's resilience and vulnerability in Haitian [12] and anxiety in cancer patients [13]. However, studies on S-HTP drawing test and depression are rarely found in cancer patients. Therefore, the study of S-HTP drawing test and depression in cancer patients is a pioneering field in psychology research. This study was designed to investigate its applicability and develop a predictive value that identifies depression in cancer patients.

## 2. Patients and Methods

### 2.1. Study Design and Population

The cross-sectional study was conducted among 200 participants at the Second Xiangya Hospital of Central South University in Changsha, Hunan Province, China, from December 2015 to December 2017, who agreed to participate in the study. Thirty-three patients were excluded from the study because they failed to complete the self-rating depression scale (SDS) or S-HTP drawing test, and 167 cancer patients were finally included.

### 2.2. Inclusion and Exclusion Criteria

The inclusion criteria were as follows: (1) age ≥ 16 years old, (2) cancer pathology and under treatment for cancer, (3) having reading comprehension ability, and (4) consent to participate in the study. The exclusion criteria included cancer patients with severe organ dysfunction and poor physical condition who could not complete the test. Patients with a professional basis in drawing and those familiar with the rules of the drawing test were not included. In addition, patients who were under treatment for depression were excluded.

### 2.3. Procedures

All patients underwent detailed face-to-face clinical interviews. Self-edited general condition questionnaire, SDS, and S-HTP drawing test were used in the interview. The participants first did the S-HTP drawing test. After that, these cancer patients were allowed for 5 to 10 minutes to rest. Then, the self-edited general information questionnaire and SDS were fulfilled.

### 2.4. Self-Edited General Information Questionnaire

7 items including age, gender, nation, education, marital status, occupation, and income consisted of the questionnaire (Table 1).

### 2.5. Self-Rating Depression Scale (SDS)

The SDS [14] is a 20-item, self-administered, short scale that evaluates affective, physiologic and psychological symptoms of depression. It includes 10 positive-scoring items and 10 reverse-scoring items that are rated from one to four. The raw score for each of the 20 items was added together to get the total score, which multiplied by a factor of 1.25 is the standard score of SDS. According to Zung's severity levels classification criteria, the SDS score greater than 53 was used by Chinese psychiatric professionals as a cutoff point for depression-related symptom severity when conducting assessments. The higher score means severe depression status. Zung's self-rating depression scale and the cutoff values have been widely used in Chinese depression studies, and the Chinese versions of these surveys have also been validated [15, 16].

### 2.6. The S-HTP Drawing Test Applied

In our previous published study, the whole process of S-HTP drawing test was described in detail [13]. All of the patients were supplied with an A4 sheet of paper, a 2B-pencil, a writing board, and an eraser. They were asked to draw a house, a tree, a person and anything you want on the paper. If they wished, they could change the drawing by using the eraser. There was no set time for them to complete their drawing but the test must be completed by themselves.

### 2.7. S-HTP Drawing Test Scoring System

Based on R.C. Burns' drawing test interpretation system and other relevant literature, we identified a set of drawing features related to depression [17–19]. A total of 23 drawing features of depression were identified. These items were subsequently divided into four areas of painting characteristics. (Table 2). First, overall S-HTP features included D1~D6; Second, house features included D7~D10; Third, tree features included D11~D16. Finally, person features included D17~D23. If certain characteristics were found to be present, each item was assigned 1 score, otherwise, it was 0 score. The assessment of the drawing characteristics was carried out independently by two professionally trained researchers, and if the assessment is controversial, it is decided by a third professional.

### 2.8. Reliability and Validity Evaluation

The intertester reliability and test-retest reliability were used to evaluate the reliability of S-HTP test drawing features in this research. Intertester reliability and test-retest reliability are expressed by the Kappa coefficient and correlation coefficient r, respectively. The value of the Kappa coefficient ranged from 0.752 to 1.000, and r of 20 patients retested was 0.710 to 0.857, which means the results of the drawing features were stable and reliable. In order to represent the S-HTP drawing test validity, the criterion validity was used in this study. The data of depression from cancer patients identified by S-HTP drawing test and SDS scale was used to perform the correlation analysis. The result of SDS scale was regarded as the “gold standard” in this study, and the validity coefficient was regarded as the correlation coefficient.

### 2.9. Statistical Analysis

The data were analyzed with SPSS version 19 for Windows (SPSS Inc., Chicago, IL, USA). Single-sample Kolmogorov-Smirnov test was used to test whether the continuous variables are normally distributed. The mean ± SD (standard deviation) was used to represent the continuous variables which conform to the normal distribution, and the median (interquartile range [IQR]) was used to represent the nonnormal variables. Two independent samples Student's t-test were used to compare the mean of two continuous variables which conform to normal distribution. Mann-Whitney U test and Kruskal-Wallis test were used to compare the mean values of 2 groups, 3 groups or more groups of nonnormal distribution variables. The frequencies of categorical variables were compared using Pearson *χ*2 or Fisher's exact test, when appropriate. The role of S-HTP drawing characteristics in predicting depression was examined with the logistic regression equation. A value of* P* less than 5% is of statistical significance.

## 3. Results

### 3.1. General Characteristics of Cancer Patients

Initially, a total of 200 patients were enrolled in the study, and 33 cases of them were excluded due to missing questionnaires or drawings. Finally, 167 patients with cancer were included in the study, and the effective rate was 83.5%. Among the 167 cancer patients, 98 patients were male, accounting for 58.7%, and 69 were female, accounting for 41.3%. Their age ranged from 16 to 72 years, with an average age of (52.92 ± 10.43) years. The general characteristics of all patients are shown in Table 1.

### 3.2. The Incidence of Depression among Cancer Patients

The score of SDS ranged from 25.00 to 80.00, with an average of (42.88 ± 10.43) in this study. Compared to the norm standard score [20] (The SDS norm standard is the result of Wang's evaluation of 1340 normal Chinese people, where the standard score is (41.85 ± 10.57)), the difference was statistically significant (t=8.708, P<0.001). Fifty-seven people achieved a diagnostic score of depression of over 53 points and 110 patients did not. The incidence rate of depression among these cancer patients was 34.1% and nondepression was 65.9%.

### 3.3. The Incidence of Various Items Used in Depression Assessment in Drawing Characteristics and Univariate Analysis

The incidence of the relevant evaluation items is shown in Table 2 according to the drawing characteristics of the cancer patients. Among the items, those with a higher incidence of depression status were D8, D17, D20, D4, and D5. Considering the number of S-HTP drawing features (depression dimensions) and the number of subjects in this study, we performed a univariate analysis based on statistical theory. Chi-square test was performed on all 23 items in the depression and nondepression groups. The results showed that there were 11 different characteristics in the depression group compared with the nondepression group. In the logistic regression analysis, to capture all important factors and avoid the relationship between some independent variables and dependent variables being masked due to confounding factors, the characteristics with a* P* value of less than 0.15 were used as independent variables. The details of these drawing features are shown in Table 2.

### 3.4. Logistic Regression Analysis

In this study, 11 depressive drawing features (two categorical variables, selected by univariate analysis) were used as independent variables, and the results of the SDS (setting the depression group to 1 and the depression-free group to 0) were used as the dependent variable, then, a logistic regression model was established (*α*_in_ = 0.10, *α*_out_ = 0.15). To explore the relationship between the characteristics of S-HTP drawing test and depression in cancer patients, a total of 9 drawing features were included in the equation. The total test results of the model coefficients revealed that *χ*2=68.657, P < 0.001, indicating that the established logistic regression equation model was statistically significant. Based on the regression coefficients shown in Table 3, a regression equation for the characteristics of the S-HTP drawing test on depression in cancer patients was established. Subsequently, the Nagelkerke R2 coefficient test was performed on the established regression equation. The regression equation established was as follows: Logit(P)= -2.997+1.345*∗*D1+0.919*∗*D2+2.044*∗*D3-0.888*∗*D5-0.944*∗*D6+1.439*∗*D9+2.106*∗*D10+0.679*∗*D14+1.148*∗*D23. The Nagelkerke R^2^ value was determined to be 0.466, showing good fitness. These results indicate that the equation established in this study have a good level of interpretation, and the S-HTP drawing features were suitable for the assessment of depression in cancer patients.

### 3.5. The Results of S-HTP Drawing Test for Depression in Cancer Patients and Its Validity

In 57 depression patients with cancer who were diagnosed by the SDS scale, the logistic regression equation was employed to predict depression. The results showed that 32 patients with cancer had depression, and the correct rate was 56.1% (32/57). At the same time, the logistic regression equation was performed in 110 cancer patients without depression, and the finding demonstrated that 98 cancer patients did not have depression, with a correct rate of 89.1% (98/110). The total correct rate was 77.8% (130/167), Table 4. A correlation analysis was performed between the results of the S-HTP drawing test and SDS scale in order to verify the validity of the S-HTP drawing test for depression in cancer patients. The test results of the correlation coefficient of two detection methods $r=\left(ad-bc\right)/\sqrt{\left(a+b)(c+d)(a+c)(b+d\right)}=\left(32\times 98-25\times 12\right)/\sqrt{57\times 110\times 44\times 123}$ =0.49, r>0.40, indicated good consistency between S-HTP drawing test results and SDS results. Further consistency check was performed, *χ*^2^=[(|*ad*-*bc*| -n/2)^2^n]/[(*a*+*b*)(*c*+*d*)(*a*+*c*)(*b*+*d*)]=[(|32 × 98-25 × 12| -167/2)^2^ ×167]/(57×110×44×123)=37.29. *χ*^2^>6.63,* P*<0.01 ($\overset{2}{\chi^{0.01,1}}$=6.63). We found that the results of the S-HTP drawing test for depression in cancer patients were positively correlated and consistent with those of the SDS scale.

### 3.6. Univariate Analysis of SDS Scores in Different Subgroups

Univariate analysis of SDS scores in different subgroups was performed as the depression levels vary with a wide range of other factors such as types of cancer, metastasis, pain degree, comorbidity disease and stage of the tumor. The results showed that SDS scores were higher in cancer metastasis group (*p* < 0.05), and there were no differences in SDS scores in other subgroup analyses (*p *> 0.05) (Table 5).

## 4. Discussion

Depression refers to a painful experience when an individual feels sad, distressed or discouraged. Its characteristic symptoms include the disappearance of pleasure, helplessness, despair, self-guilt and suicidal tendencies [21]. Depression has become the most common negative emotion in cancer patients [22]. The average SDS standard score of cancer patients was 48.88±10.43 points, which was significantly different from that of the norm (t=8.708, P<0.001). Among them, 57 (34.1%) met the diagnostic criteria of depression. There was a significant difference in the score of SDS between cancer patients with or without metastasis (t = 2.032, P=0.044). The metastasis means the severity of the disease, which has a significant impact on the depression state in cancer patients.

Most human thoughts and mental activities can be visualized. For example, the presence of physical objects can significantly enhance memory. Therefore, mental problems, such as depression can be better identified and resolved by analyzing drawings [13]. S-HTP drawing test provides an important source of clinical information. They have been a valuable part of test batteries for clinicians and psychologists over the years, seen as revealing important aspects of an individual's personality in a drawing [13]. They provide an understanding of, and insight into, the individual through creative expression of raw emotion. S-HTP drawing test has routinely uncovered unconscious determinants of self-expression that possibly could not be manifested in direct communication [23]. It is believed that verbal communication is more subject to conscious manipulation than graphics projection. In many cases, drawings have revealed the individual's emotional, depression, anxiety, and even schizophrenia. Therefore, drawing can serve as an avenue for such individuals to express themselves.

The five items with a high incidence of depression assessment items were D8, D17, D20, D4, and D5, suggesting that low mental motivation, pessimistic about real life and melancholy are prevalent in cancer patients. This study found that the correlation coefficient between the result of S-HTP drawing test and SDS was 0.49, and the consistency hypothesis test showed that S-HTP drawing test was positively correlated with SDS. These results showed that S-HTP drawing test was feasible and effective in evaluating depression in patients with cancer. This can also be proved by the logistic regression equation established to screen depression. The nine painting features included in the regression equation were D1, D2, D3, D5, D6, D9, D10, D14, and D23. The detail explanation and psychological meaning of these items are shown in Table 6. It is noteworthy that many drawing evaluation items used in this study were not included in the regression equation. The main reason may be that the characteristics of this drawing were low frequency or difficulty in comprehension. Even though these S-HTP drawing test items may be related to depression, their predictability is less significant compared to the drawing characteristics items that correlate with the logistic regression equation.

The occurrence of depression in cancer patients could be attributed to the diagnosis of cancer itself, the heavy economic burden of treatment and poor therapeutic effect. Depression often results in severe consequences if it is not detected early and managed effectively in cancer patients. Therefore, it is necessary to monitor the occurrence of depression in cancer patients and to establish valid evaluation methods. The S-HTP drawing test can effectively reveal the inner emotion of cancer patients because it can be performed with less psychological defense as opposed to using the questionnaire alone. In addition, the S-HTP drawing test is a novel concept which can be an effective way of communication for researchers and patients who are unwilling or unable to express their feelings verbally. The S-HTP drawing test will have more application prospects with the further standardization and improvement of modern technology in the future.

There are some limitations in this study. First, the study cannot find causal explanations because of its cross-sectional characteristic. Second, this study used the Zung's self-rating scale to screen for depression in cancer patients with reference to the Chinese National Normative Scores. However, the results of the SDS, drawn from self-rating scales, lack comparability to some extent with the clinical diagnostic criteria for depression disorders found in the International Classification of Diseases 10 (ICD10) and Diagnostic and Statistical Manual of Mental Disorders IV (DSM-IV). Third, the different capacity of the S-HTP drawing test to identify more the nondepressed patients than depressed even if there is an interrater and test-retest reliability between S-HTP drawing test and SDS. The clinician should clearly know the capacity of the test to distinguish patients to be pharmacologically treated or not. In addition, we did not perform cognitive function assessment on the subjects before starting this study. If the patient has cognitive impairment, it may affect the outcome. But we only included the patients who had reading comprehension ability. Finally, it is unclear whether studies on other nationalities will yield similar findings for the reason that these findings are based on Chinese patients.

## 5. Conclusion

S-HTP drawing test and SDS have interrater reliability and test-retest reliability. Our findings indicate that the S-HTP drawing test could help in screening depression in cancer patients.

## Figures and Tables

**Table 1:** Demographic characteristics of cancer patients.

Items	Characteristics	Cases	Constituent ratio (%)
Age(years)	<45	40	24.0
	45-60	83	49.7
	>60	44	26.3

Gender	Male	98	58.7
	Female	69	41.3

Nation	Han	161	96.4
	minority	6	3.6

Education	Elementary or junior	98	58.7
	Senior	44	26.3
	College or above	25	15.0

Marital status	Married	155	92.8
	Unmarried	12	7.2

Occupation	Civil servant or teacher	16	9.6
	Worker	22	13.2
	Farmer	71	42.5
	Businessman or freelancer	24	14.4
	Retired or unemployed	34	24.4

Income of the family per month (Ren Min Bi)	<1000	22	13.2
	1000~1999	76	45.5
	2000~2999	55	32.9
	3000~3999	10	6.0
	>4000	4	2.4

**Table 2:** The incidence of items used in depression assessment and drawing characteristics in depression group and in nondepression group of cancer patients.

Drawing characteristics		Cases	Depression self-evaluation results		*χ* ^2^ value	*P *value
			Depression	No depression		
D1 Small drawing size	Yes	46 (27.5)	27 (47.4)	19 (17.3)	17.039	<0.001
	No	121 (72.5)	30 (52.6)	91 (82.7)		

D2 Weak strength drawing	Yes	47 (28.1)	23 (40.4)	24 (21.8)	6.376	0.012
	No	120 (71.9)	34 (59.6)	86 (78.2)		

D3 Simplified drawing	Yes	96 (57.5)	44 (77.2)	52 (47.3)	13.753	<0.001
	No	71 (42.5)	13 (22.8)	58 (52.7)		

D4 Scribbled drawing	Yes	130 (77.8)	80 (80.8)	50 (73.5)	1.238	0.266
	No	37 (22.2)	19 (19.2)	18 (26.5)		

D5 Decorated drawing	Yes	107 (64.1)	30 (52.6)	77 (69.1)	4.387	0.036
	No	60 (35.9)	27 (47.4)	33 (30.9)		

D6 Lines jagged and not joined	Yes	86 (51.5)	23 (40.4)	63 (57.3)	4.304	0.038
	No	81 (48.5)	34 (59.6)	47 (42.7)		

D7 Simple house drawing	Yes	67 (40.1)	42 (42.9)	25 (36.2)	0.740	0.390
	No	100 (59.9)	56 (57.1)	44 (63.8)		

D8 Simple house	Yes	136 (81.4)	51 (81.0)	85 (81.7)	0.016	0.900
	No	31 (18.6)	12 (19.0)	19 (18.3)		

D9 Decorated house	Yes	57 (34.1)	27 (47.4)	30 (27.3)	6.744	0.009
	No	110 (65.9)	30 (52.6)	80 (72.7)		

D10 Very small door	Yes	18 (10.8)	14 (24.6)	4 (3.6)	17.095	<0.001
	No	149 (89.2)	43 (75.4)	106 (96.4)		

D11 Simple tree drawing	Yes	85 (50.9)	60 (52.6)	25 (47.2)	0.432	0.511
	No	82 (49.1)	54 (47.4)	28 (52.8)		

D12 Simple tree	Yes	43 (25.7)	22 (38.6)	21 (19.1)	7.472	0.006
	No	124 (74.3)	35 (61.4)	89 (80.9)		

D13 Decorated tree	Yes	63 (37.7)	26 (40.6)	37 (35.9)	0.372	0.542
	No	104 (62.3)	38 (59.4)	66 (64.1)		

D14 Dead tree	Yes	54 (32.3)	24 (42.1)	30 (27.3)	3.775	0.052
	No	113 (67.7)	33 (57.9)	80 (72.7)		

D15 Scribbled or carefully drawn tree-crown	Yes	69 (41.3)	42 (38.9)	27 (45.8)	0.744	0.389
	No	98 (58.7)	66 (61.1)	32 (54.2)		

D16 Slender tree trunk	Yes	55 (32.9)	30 (31.6)	25 (34.7)	0.183	0.669
	No	112 (67.1)	65 (68.4)	47 (65.3)		

D17 Simple person drawing	Yes	135 (80.8)	82 (82.0)	53 (79.1)	0.217	0.641
	No	32 (19.2)	18 (18.0)	14 (20.9)		

D18 Simple person	Yes	56 (33.5)	26 (31.7)	30 (35.3)	0.241	0.624
	No	111 (66.5)	56 (68.3)	55 (64.7)		

D19 Decorated person	Yes	53 (31.7)	36 (30.5)	17 (34.7)	0.280	0.597
	No	114 (68.3)	82 (69.5)	32 (65.3)		

D20 Poker face	Yes	135 (80.8)	57 (85.1)	78 (78.0)	1.296	0.255
	No	32 (19.2)	10 (14.9)	22 (22.0)		

D21 Complete or partial loss of facial features	Yes	95 (56.9)	39 (68.4)	56 (50.9)	4.695	0.030
	No	72 (43.1)	18 (31.6)	54 (49.1)		

D22 Complete or partial loss of limbs	Yes	46 (27.5)	22 (28.2)	24 (27.0)	0.106	0.745
	No	121 (72.5)	56 (71.8)	65 (73.0)		

D23 Head drew very carefully	Yes	60 (35.9)	25 (43.9)	35 (31.8)	2.365	0.124
	No	107 (64.1)	32 (56.1)	75 (68.2)		

**Table 3:** Logistic regression analysis examining the role of S-HTP drawing characteristics in predicting depression.

Included painting characteristics	*b*	*S*_*b*_	Wald*χ*^2^	*P*	OR	95%OR	
						Lower	Upper
D1	1.345	0.495	7.393	0.007	3.839	1.456	10.125
D2	0.919	0.492	3.482	0.062	2.506	0.955	6.579
D3	2.044	0.515	15.723	<0.001	7.719	2.811	21.198
D5	-0.888	0.486	3.341	0.068	0.411	0.159	1.066
D6	-0.944	0.450	4.407	0.036	0.389	0.161	0.939
D9	1.439	0.481	8.960	0.003	4.215	1.643	10.810
D10	2.106	0.790	7.110	0.008	8.218	1.747	38.647
D14	0.679	0.439	2.389	0.122	1.972	0.834	4.666
D23	1.148	0.490	5.493	0.019	3.151	1.207	8.229
Constant	-2.997	0.689	18.890	<0.001	0.050		

**Table 4:** Comparison of the incidence of depression in cancer patients by SDS and S-HTP.

SDS	S-HTP test		Total
	Depression	No depression	
Depression	32	25	57
No depression	12	98	110

Total	44	123	167

**Table 5:** Univariate analysis of SDS scores in different subgroups.

Items	Cases	Score ($\overset{-}{\chi }\pm s$)	Test-value	*P* value
Type of Cancer				
leukemia	2	51.25±0.00	H=14.680	0.198
bladder cancer	1	46.25		
nasopharyngeal carcinoma	27	49.54±10.74		
lung cancer	85	48.12±10.35		
cervical cancer	15	55.17±11.81		
colorectal cancer	13	47.79±7.94		
lymphoma	6	40.63±5.63		
ovarian cancer	2	48.50±0.00		
breast cancer	3	61.25±17.32		
esophageal cancer	6	42.50±7.75		
gastric cancer	4	53.75±10.10		
malignant thymoma	3	47.50±6.50		
Metastasis				
yes	54	51.23±9.59	t=2.032	0.044
no	113	47.75±10.67		
Pain degree				
none	88	47.98±9.96	F=0.734	0.533
mild	55	49.30±10.54		
moderate	20	50.69±12.29		
serious	4	53.75±10.10		
Comorbidity disease				
yes	42	47.35±12.11	t′= -0.988	0.327
no	125	49.39±9.80		
Tumor stage				
I	5	54.00±9.41	F=1.771	0.137
II	10	54.25±15.37		
III	45	47.97±10.23		
IV	53	50.17±9.33		
undetermined	54	46.90±10.36		

**Table 6:** Explanation and psychological meaning of major S-HTP drawing features.

Drawing features	Explanation	Psychological meaning
D8 Simple house	No other description on the house except for the basic features of walls, doors, and windows	Avoidance and pessimistic psychology, and depression

D17 Simple person drawing	Absence of particular detail	Avoidance and pessimistic psychology, and depression

D20 Poker face	No expression on the face	Avoidance and pessimistic psychology, and depression

D4 Scribbled drawing	The painting was scribbled	Poor emotional control and inner distress

D5 Decorated drawing	Other embellishments to the drawing besides house, tree, and person, such as sun, flowers, lawns, animals, etc	Experienced lonely and depression, eager for social support, care and accompany

D1 Small drawing size	The size of the drawing is less than 1/3 compared with the whole paper area	Inferiority complex and self-inhibition, a tendency to regression, and an absence of mental drive

D2 Weak strength drawing	The strength of lines is weak	Lack of mental motivation and self-confidence, bad mood, the inner energy is missing and the ability of self-control is weak

D3 Simplified drawing	Use of a single line or outline in the drawing	Avoidance psychology

D6 Lines jagged and not joined	Lines jagged and more than two breaks on one line	Innermost feelings are very fragile

D9 Decorated house	The house is decorated, as in addition to the house, but also painted sidewalks, etc	Depressed and lonely, and hope to get more social support, attention, and accompany

D10 Very small door	The door is less than 1/4 of the wall	Introverted and withdrawn

D14 Dead tree	No tree-crown or no leaves on the branches	Low psychological energy level

D23 Head drawn very carefully	Emphasizes hairstyle, hair ornament and hat on head	Inner fantasy

## Data Availability

The data used to support the findings of this study are available from the corresponding author upon request.
